# Elasmobranchs from Indonesian Waters: Feeding Ecology and Trypanorhynch Cestode Fauna Composition to Support Efforts in Shark and Ray Conservation

**DOI:** 10.1007/s11686-022-00593-7

**Published:** 2022-09-08

**Authors:** S. Kleinertz, I. Yulianto, C. Kurschat, S. Koepper, B. M. Simeon, S. Klimpel, S. Theisen, P. Unger, H. Retnoningtyas, X. Neitemeier-Duventester, D. P. Barton, I. M. Damriyasa, H. W. Palm

**Affiliations:** 1 Aquaculture and Sea-Ranching, Faculty of Agricultural and Environmental Sciences, University of Rostock, Justus-von-Liebig-Weg 2, 18059 Rostock, Germany; 2 Faculty of Fisheries and Marine Sciences, Bogor Agricultural University, Jl. Agatis Kampus IPB Dramaga, Bogor, Indonesia; 3 Fisheries Resource Center of Indonesia (FRCI), Jl. Sempur No. 35, Bogor, West Java 16129 Indonesia; 4 Institute of Zoomorphology, Cell Biology and Parasitology, Heinrich-Heine-University Düsseldorf, Düsseldorf, Germany; 5 University of Bremen, Bibliothekstr. 1, 28359 Bremen, Germany; 6 Institute for Ecology, Evolution and Diversity, Biodiversity and Climate Research Centre (BiK-F) Goethe-University, Frankfurt am Main, Germany; 7 School of Agricultural, Environmental and Veterinary Sciences, Charles Sturt University, Wagga Wagga, New South Wales 2651 Australia; 8 Centre for Studies on Animal Diseases, Faculty of Veterinary Sciences, Udayana University Bali, Badung Denpasar Bali, 80363 Indonesia

**Keywords:** Shark and ray conservation, Parasites of elasmobranchs, Cestode community

## Abstract

**Purpose:**

The stomachs and spiral valves of sharks and rays were examined for their trypanorhynch (Cestoda) parasite fauna and dietary items to infer feeding ecology. In Indonesia, sharks and rays have been experiencing increasing awareness and conservation in the recent years due to high fisheries activities and to avoid future species extinction.

**Methods:**

The samples were collected in 2009 from two different sampling sites at the southern coasts of Java and Bali in Indonesia. The parasite fauna was studied for 41 elasmobranch fishes. Amongst these, three shark species, *Carcharhinus sorrah*, *Carcharhinus* sp. I and *Squalus megalops* and seven ray species, *Brevitrygon heterura, B.* cf. *heterura, Gymnura zonura, Maculabatis gerrardi, Mobula kuhlii*, *Neotrygon cauruleopuncatata* and *Rhinobatos penggali* were studied. Four additional specimens, belonging to the shark species *Carcharhinus* sp. II and *Mustelus* cf. *manazo* and the ray species *Maculabatis gerrardi* were studied from the waters of South Bali.

**Results:**

Analyses of the feeding ecology of the ray *M. gerrardi* revealed distinct differences between both sampling sites, indicating the presence of ecological differences between the geographically independent regions. A total of 11 different trypanorhynch species/taxa belonging to the five families Eutetrarhynchidae (5), Gilquiniidae (1), Lacistorhynchidae (1), Pterobothriidae (1) and Tentaculariidae (3) were found. Ten trypanorhynch species from Penyu Bay and four species from South Bali could be identified. Two taxa that might represent new species were collected: *Dollfusiella* sp. from *Brevitrygon heterura* and *Prochristianella* sp. from *Maculabatis gerrardi.*

**Conclusions:**

The present paper gives insights in using the trypanorhynch cestode community in combination with feeding ecology analyses to support conservation of elasmobranchs in Indonesian waters.

## Introduction

With more than 200 species of sharks and rays, Indonesia is considered to have one of the richest elasmobranch faunas in the world. However, it is also a region where shark and ray populations are amongst the most heavily exploited with a reported 105,000 and 118,000 tonnes landed in 2002 and 2003, respectively [1–3]. Elasmobranchs are caught within Indonesian waters by both target fisheries and as bycatch by both small-scale fishers and commercial operators. A variety of fishing methods are used in the target fisheries, such as gill and tangle nets, longlines and harpoon [3]. Fisheries that land substantial catches of elasmobranchs as bycatch, include those operating bottom trawls, trammel and gill nets, longlines and droplines [1–3]. Available biological data or information concerning size compositions of species landed are scarce [3, 4]. The taxonomic and ecological knowledge of Indonesia’s elasmobranch fauna needs improving to provide an adequate baseline for data acquisition and resource management [3].

## Ecology of Sampled Elasmobranchs

As apex predators, elasmobranchs play an important role in the marine food-web. Sharks and rays prey on a large range of organisms and therefore reductions in their population size can initiate trophic cascades through top–down effects [5–7]. Their diet includes crustaceans, cephalopods, bony fish (Teleostei) and other elasmobranch species (e.g., *Squalus megalops*; [18]). When considering their broad distribution and their extensive diet range, elasmobranchs are a crucial component in the energy transfer and food-web dynamics of the ocean [22–24].

The total world catch from wild marine stocks has increased in the recent years [8]. Fishing pressure and habitat loss have resulted in substantial declines in shark populations [5, 7, 9] resulting in approximately 40% of shark species being threatened with extinction [10]. Elasmobranchs show a high vulnerability to overfishing and other threats, such as habitat loss, due to their slow life cycle [11–14]. Nearly all of the examined shark and ray species within this study have, as do most elasmobranchs, an ovoviviparous reproduction (e.g., *Squalus montalbani*) where the embryos remain in the mother’s body until they are ready to hatch [15, 16]. Gestation periods up to 2 years (e.g., *S. montalbani*; [17]), sexual and geographical segregation of sexes (e.g., *Scymnodon plunket*; [12, 18, 19]) and late maturity (e.g., 22 years in the female *Centroscymnus creptidater*; [20]) are important factors that contribute to the relatively low reproductive rate of these cartilaginous fishes [21].

According to the IUCN Red List of Threatened Species [25], the conservation status of elasmobranch species that were examined in this study ranges from near threatened (*Brevitrygon heterura*) to vulnerable (*Rhinobatos penggali*) (Table 1). The government of Indonesia protect 11 species of elasmobranchs, i.e., whale shark (*Rhincodon typus*), manta ray (*Mobula alfredi* and *M. birostris*), freshwater rays (*Fluvitrygon oxyrhincha, F. signifier* and *Urogymnus polylepis*) and saw fish (*Anoxypristis cuspidata* and *Pristis* spp.). Additionally, they regulate the fishing of several species listed under CITES, including an export ban for thresher shark (*Alopias* spp.) and quotas for hammerhead sharks (*Sphyrna* spp.). Moreover, a few provincial governments have been developing initiatives to regulate fishing effort on sharks to control fishing mortality.

**Table 1:** Conservation status of elasmobranch species that were examined in the present study (https://www.iucnredlist.org/, downloaded 18 January 2022)

Species	IUCN Red List status	Distribution
*Brevitrygon heterura* (R)	Vulnerable	All Indonesian waters
*Gymnura zonura* (R)	Endangered	All Indonesian waters
*Maculabatis gerrardi* (R)	Endangered	All Indonesian waters
*Mobula kuhlii* (R)	Endangered	All Indonesian waters
*Neotrygon caeruleopunctata* (R)	Least concern	Indonesian waters, Java Sea to the edge of the Indian Ocean
*Rhinobatos penggali* (R)	Endangered	Southern Indonesian waters
*Carcharhinus sorrah* (S)	Near threatened	All Indonesian waters
*Mustelus* cf. *manazo* (S)	Not assessed	Southern Indonesian waters, possibility endemic in Indonesia
*Squalus megalops* (S)	Vulnerable	Southern Indonesian waters

*R* ray species, *S* shark species

## Elasmobranch Health

Questions concerning protection, conservation and the general health status of endangered elasmobranchs, have risen frequently in the recent years. As many elasmobranch species are endangered, their health status needs to be especially focussed on for future conservation (Table 1). Elasmobranchs are difficult to observe in nature and information on rare or less-frequently caught species is scarce [26]. This particularly concerns aspects of their ecology, main habitat, migration patterns, depth range and most important prey items [26] as well as health status and parasitism. According to Palm [27], fish parasites have been widely used as biological indicators for the host ecology, but the methods applied for the elasmobranchs are, so far, limited due to often restricted and unpredictable catches and less availability of specimens to study [26]. Parasitic diseases are increasingly recognized for their profound influences on individual, population and even ecosystem health [28]. Elasmobranchs have been reported as hosts of parasitic species, especially belonging to the order Trypanorhyncha Diesing, 1863, members of the Cestoda [26].

According to Palm [29], the Trypanorhyncha are distributed worldwide and amongst the most species-rich orders of marine cestodes. They infect stomachs and intestines of elasmobranchs as final hosts. Their larval stages occur in a wide range of organs of teleost fishes and a variety of marine invertebrates [29].

## Study Areas

The city of Cilacap in Penyu Bay, South Central Java is surrounded by the brackish Segara Anakan lagoon, and is characterized by an oil refinery plant, a deep-sea harbour, a cement and a fertilizer factory. The lagoon plays an important role as a mating-, breeding- and nursery ground for a vast number of aquatic organisms. It is the last extensive mangrove system on Java [30]. The lagoon is impacted by sedimentation and deforestation [31], with pollution like heavy metals, pesticides, hydro carbonates and sediments [32] and minor to moderate nutrient pollution and eutrophication [33]. Although most coasts of the Indonesian archipelago have direct access to the deep-sea (below 200 m), the coastline of Penyu Bay, is comparatively shallow. According to Palm and Rückert [30, 34], the Segara Anakan lagoon has underlying inconsistent, varying environmental influences, including the change of rainy- and dry season, sedimentation and turbidity and differences in salinity. Fisheries constitute a main part of the local economy. The most important commercial fish species in the fish markets in Cilacap are tunas, sharks and rays. Inside the lagoon, many economically important species, such as milkfish and grouper, are farmed in aquaculture facilities.

The Bay of Kedonganan is located at the western side of the southern tip of Bali, directly next to the Ngurah Rai Kuta International Airport. The airstrip of the airport reaches into the ocean and acts as the northern border of the bay. There is no harbour; the small ships lay directly in front of the beach which is used to land captures. The fishermen catch fish from the Bali Strait and from areas close to South Bali and East Java. Kedonganan is a fishing village but is heavily influenced by tourism. A cooperation of the local fishermen manages the market [35]. In the fish market in Kedonganan, scombrids (tunas, mackerels and bonitos) as well as sharks are the most important species. Additional fishes imported from other Indonesian regions are also available.

The present study aimed to identify the trypanorhynch community of elasmobranchs from the southern Balinese and Javanese coasts. The relationship of the recorded parasite fauna with the elasmobranch feeding ecology is discussed, including the vulnerable (e.g., *Brevitrygon heterura, Squalus megalops*) to endangered (e.g., *Gymnura zonura, Rhinobatos penggali*) classified shark and ray species from Indonesian waters (Table 1).

## Material and Methods

### Sample Collection

The parasite fauna of 45 elasmobranch specimens was studied from the primary sampling site at Penyu Bay, South Central Java, in 2009 (Table 2, Table 3). Amongst these were the three shark species *Carcharhinus sorrah* (Carcharhinidae, Jordan & Evermann, 1896)*, Carcharhinus* sp. I (Carcharhinidae), and *Squalus megalops* (Squalidae, Blainville, 1816)*,* and the seven ray species *Brevitrygon heterura* (Dasyatidae, Jordan & Gilbert, 1879)*, B.* cf. *heterura* (Dasyatidae)*, Gymnura zonura* (Gymnuridae, Fowler, 1934), *Maculabatis gerrardi* (Dasyatidae) and *Rhinobatos penggali* (Rhinobatidae, Bonaparte, 1835)*, Mobula kuhlii* (Myliobatidae, Bonaparte, 1835) and *Neotrygon caeruleopunctata* (Dasyatidae). The two shark species *Carcharhinus* sp. II (Carcharhinidae) and *Mustelus* cf. *manazo* (Triakidae, Gray, 1851) and the ray species *M. gerrardi* (Dasyatidae) were studied from the waters of South Bali in the same year.

**Table 2:** Biological and catch data of investigated ray species from different locations in 2009

Species	Location	*n*	TL (cm)	BL (cm)	Width (cm)	TW (g)	W SV (g)	W ST full (g)	W ST empty (g)	*m*	*f*	n.i.
*Brevitrygon* *heterura*	PB	5	42.8	20.3	19	255.9	2.9	4.2	2.5	1	2	2
			(36.0–53.0)	(18.5–24.0)	(17.0–22.0)	(175.6–396.6)	(1.7–4.4)	(3.0–4.9)	(2.0–3.0)			
*Brevitrygon* cf.* heterura*	PB	5	53.2	18.4	18.4	215.5	3.8	3	2	4	1	–
			(45.0–57.0)	(17.0–20.0)	(17.0–19.0)	(177.4–248.6)	(1.6–5.0)	(1.9–4.2)	(1.6–2.2)			
*Gymnura zonura*	PB	5	44.8	31.2	n.d.	2630	27.6	31.8	24.1	1	1	3
			(33.0–76.0)	(23.0–55.0)		(650–9000)	(10.8–74.8)	(15.3–72.3)	(11.4–63.6)			
*Maculabatis* *gerrardi*	JB	1	n.d.	59.5	55	4800	86	39.9	25.5	1	–	–
*Maculabatis* *gerrardi*	PB	5	167.9	44.8	n.d.	3980	71.4	48.1	29.1	1	–	4
			(149.0–180.0)	(40.0–52.0)		(2900–6000)	(54.1–108.0)	(23.5–74.0)	(14.9–44.3)			
*Mobula kuhlii*	PB	1	83.5	64	107.7	12,000	131.6	117.3	116.4	–	1	–
*Neotrygon caeruleopunctata*	PB	2	74	30.5	n.d.	1,300	12.9	15.8	8.6	n.d.		
			(73.0–75.0)	(30.0–31.0)		(1300–1300)	(10.8–15.0)	(15.3–16.3)	(8.3–8.9)			
*Rhinobatos penggali*	PB	5	74.8	n.d.	n.d.	1300	14.6	22.2	13.2	n.d.		
			(67.0–48.0)			(900–1600)	(11.3–17.2)	(13.2–36.8)	(6.4–22.5)			

Location: *PB* Penyu Bay (Cilacap, Java), *JB* Jimbaran (Kedonganan, Bali), *n* number of rays investigated, *TL* total length in cm (including tail), *BL* body length in cm (excluding tail), Width of body in cm, *TW* total weight in g, *W SV* weight spiral valve in g, *W ST* weight stomach (full and empty) in g, measurements as mean and range in parentheses, *m* male, *f* female, *n.i*. not identifiable, *n.d*. no data

**Table 3:** Biological and catch data of investigated shark species from different locations in 2009

Species	Location	*n*	TL (cm)	CF (cm)	PF (cm)	TW (g)	W SV (g)	W ST full (g)	W ST empty (g)	*m*	*f*	n.i.
*Carcharhinus sorrah*	PB	4	95.5	28	15.3	4175	46.3	80.9	61.2	1	3	–
			(78.0–110.0)	(21.5–32.0)	(9.5–23.5)	(2400–6500)	(37.5–64.6)	(41.5–114.7)	(34.9–84.4)			
*Carcharhinus *sp. I	PB	4	81.9	21.8	10.1	2650	36.1	118	54.9	–	3	1
			(71.5–92.0)	(19.0–24.0)	(8.5–12.0)	(1800–3500)	(12.2–49.5)	(58.7–161.7)	(45.1–65.4)			
*Carcharhinus *sp. II	JB	1	89	24	10.5	3800	66.7	52.4	38.4	–	1	–
*Mustelus* cf. *manazo*	JB	2	78	15.3	9.5	1650	25.8	30.4	18.8	2	–	–
			(63.0–93.0)	(12.0–18.5)	(7.0–12.0)	(700–2600)	(15.4–36.2)	(13.1–47.7)	(10.2–27.4)			
*Squalus megalops*	PB	5	64.3	n.d.	n.d.	1280	23.7	48.7	14.4	n.d.		
			(63.5–65.0)			(1200–1400)	(13.9–29.8)	(20.1–140.0)	(10.5–17.1)			

Location: *PB* Penyu Bay (Cilacap, Java), *JB* Jimbaran (Kedonganan, Bali), *n* number of sharks investigated, *TL* total length in cm, *CF* length of caudal fin in cm, *PF* length of pectoral fin in cm, *TW* total weight in g, *W SV* weight spiral valve in g, *W ST* weight stomach (full and empty) in g, measurements as mean and range in parentheses, *m* male, *f* female, *n.i*. not identifiable, *n.d*. no data

Fish species were identified at the markets, TPI (Tempat Pelelangan Ikan) Teluk Penyu, Cilacap, southern Java coast (Penyu Bay) and Pasar Ikan Tradisional Kedonganan, southern Bali coast (Kuta Jimbaran Bay) with relevant literature (e.g., [3, 36, 37]. Morphometric data were taken through measuring the total length (TL, anterior tip to end of tail), standard length (to beginning of tail), body width (BW, for rays only) and, for sharks, pectoral fin and caudal fin length (PFL, CFL). Photos of the habitus and various characteristics necessary for identification were taken for subsequent taxonomy. Fish weights were taken at the market by means of the official market scales. Spiral valves (stomach and intestine) were isolated by means of knifes and scissors, and were then transported on ice to the local laboratories in the Biology Faculty of UNSOED (Java) and Veterinary Faculty of UDAYANA (Bali), where they were stored in freezers at −20 °C.

## Parasitological Examination

Frozen intestinal tracts were thawed and weighed with and without contents. To release parasites from the spiral folds, the intestinal tract was cut into smaller pieces and shaken in NaCl 0.9% physiological solution in small bottles (200 ml) following the gut wash methodology of Cribb and Bray [38]. Liquids and tissues were then decanted in the dishes. Parasites were isolated from petri dishes, e.g., via further scraping off from tissue folds, under Zeiss Stemi DV4 and Olympus SZ2-ILST SZ51 binocular magnifiers.

Isolated parasites were cleaned from host tissue and gut contents and fixed in 70% EtOH, buffered with 4% formalin, and stored in 70% EtOH for subsequent analyses, e.g., microscopy. Cestoda were then stained with aceto-carmine (Mayer-Schuhberg’s, according to the protocol of Palm [29]) and mounted in Canada balsam. Drawings and photographs were prepared by means of a stereomicroscope Olympus CH-2, equipped with a Camera Lucida drawing tube (Leitz Wetzlar) and a BX50 microscope with a E410 camera (both Olympus). Scanning electron microscopy was performed with a LEO 1430 VP at the Heinrich-Heine University of Düsseldorf, after sputtering samples with gold–palladium in an argon atmosphere, according to the standard lab procedure, e.g., with 20.1 kV and different magnifications. The cestode species were identified according to Khalil *et al*. [39] and Palm [29].

Parasitological descriptors and infection rates, e.g., prevalence, (mean) abundance and (mean) intensity (P, mA, mI) were calculated according to standard procedures [40].

## Stomach Content Analysis

The stomach contents were sorted, and prey items were identified to the lowest possible taxon and grouped into broad taxonomic categories (Teleostei, Cephalopoda, Crustacea, Euphausiacea, Decapoda, Gastropoda); we grouped (combined) Crustacea, Euphausiacea, and Decapoda into Crustacea for subsequent analysis. To determine the relative importance of prey items, the numerical percentage of prey (*N*%), the weight percentage of prey (*W*%) and the frequency of occurrence (*F*%) were determined [41, 42] combined over all individuals of a species. Using these three indices, the index of relative importance, IRI [43], was calculated. The importance of a specific prey item increases with higher values for *N*, *W*, *F* and IRI.

### Statistics

Multi-variate statistical analyses were conducted with the Primer program (release 7, Primer-E Ltd. 7.0.13). Prior to the analyses, the parasite community data were fourth root-transformed to avoid an over-evaluation of rare or very frequent species. A similarity matrix was constructed using the Bray–Curtis similarity measure. Cluster analysis and non-metric multi-dimensional scaling (nMDS) were performed using the Primer software which was used to create two-dimensional ordination genera plot ([44, 45] then was clustered using group-average linking method (Field *et al*. [46])).

An one-way analyses of similarity were applied to determine the differences in community structure of parasite species composition between locations (routine ANOSIM, values close to 1 indicate high differences and close to 0 indicate high similarity between species compositions). Routine Similarity Percentage (SIMPER) analyses were applied to test which parasite species contributed most to show differences between stations. SIMPER analysis was used to determine which species was most responsible for the differences seen between sites with Bray–Curtis analysis (according to Bell and Barnes [47]).

## Results

### Fish Biological Data

**Fig. 1:**
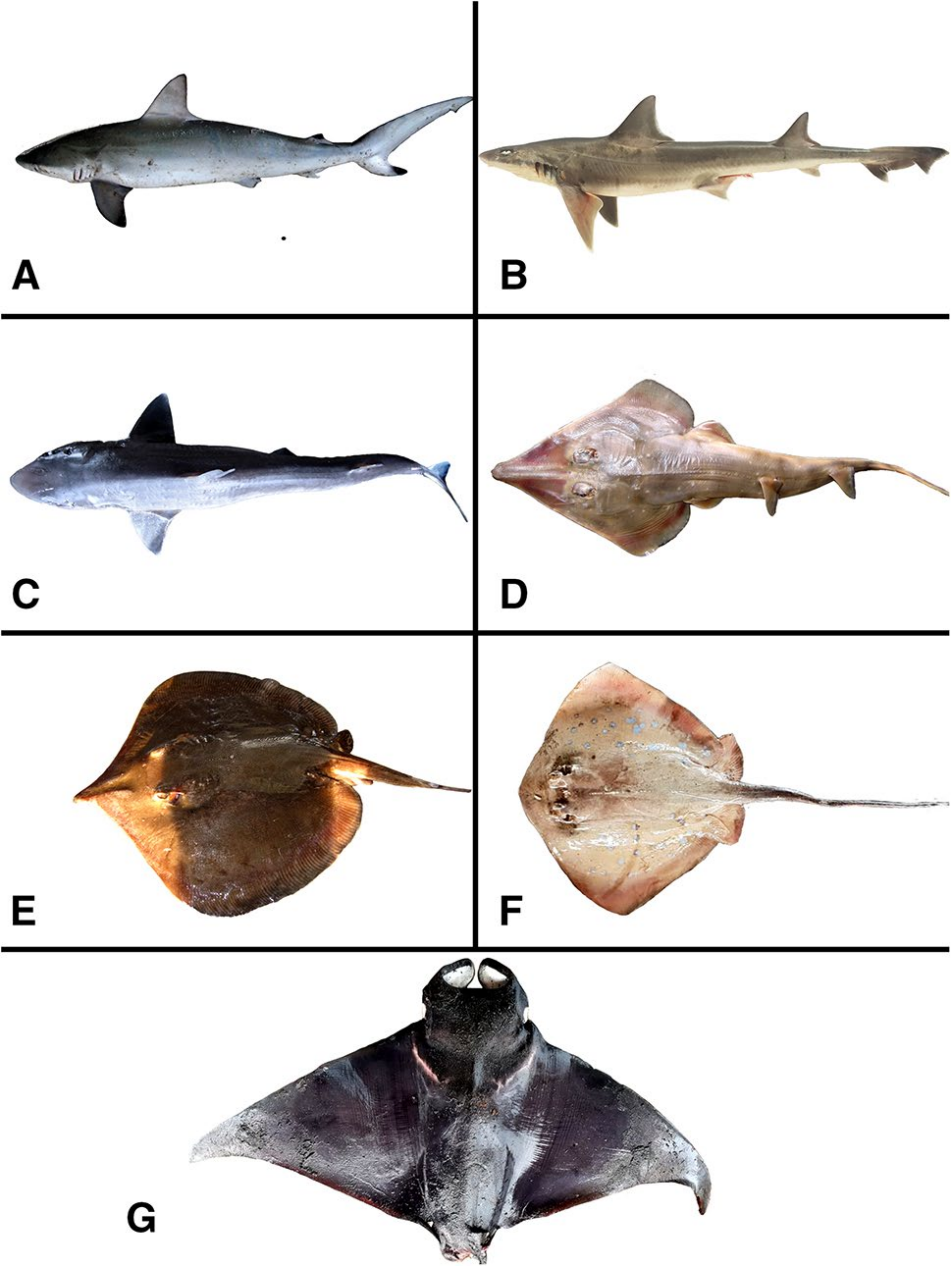
Selected examined elasmobranch species: **A** *Carcharhinus sorrah*, **B** *Mustelus* cf. *manazo*, **C** *Squalus megalops*, **D** *Rhinobatos penggali*, **E** *Brevitrygon heterura*, **F** *Neotrygon cauruleopuncatata* and **G** *Mobula kuhlii*

A total of 45 elasmobranch individuals were analysed within the present study (29 rays from seven species and 16 sharks from five species, Fig. 1).

Within the rays, *Mobula kuhlii* was the largest and heaviest species with a body length of 64.0 cm and a body weight of 12000 g. Within the sharks, *Carcharhinus sorrah* had the largest average size of 95.5 cm and the highest mean body weight of 4175 g (Table 2, Table 3).

## Stomach Contents

In general, the differences in prey items in both rays and sharks were low (Table 4, Table 5). Diet of the studied rays consisted of Teleostei, Crustacea (combined) and Gastropoda, with Crustacea being the most abundant prey item*. Brevitrygon heterura* exclusively fed on crustaceans (IRI = 16,000 and 8000, respectively), while *Maculabatis gerrardi* and *Gymnura zonura* had the most varied diet consisting of Teleostei, Crustacea and Gastropoda. The studied sharks *Carcharhinus* sp. II exclusively fed on Teleostei (IRI = 20,000). *Mustelus* cf. *manazo*, *Carcharhinus sorrah* and *Carcharhinus* sp. I had the more diverse diet and preyed upon Teleostei, Cephalopoda and Crustacea.

**Table 4:** Stomach content analyses of investigated shark species from different locations in 2009

Diet contents	*Carcharhinus sorrah*				*Carcharhinus *sp. I				*Carcharhinus *sp. II				*Mustelus *cf.* manazo*				*Squalus megalops*			
	PB				PB				JB				JB				PB			
	*F* (%)	*W* (%)	*N* (%)	IRI	*F* (%)	*W* (%)	*N* (%)	IRI	*F* (%)	*W* (%)	*N* (%)	IRI	*F* (%)	*W* (%)	*N* (%)	IRI	*F* (%)	*W* (%)	*N* (%)	IRI
Teleostei	25.0	24.1	50.0	1851.3	100.0	64.9	32.1	9706.0	100.0	100.0	100.0	20,000.0	100.0	100.0	40.0	14,000.0	100.0	95.2	88.9	18,411.0
Cephalopoda	50.0	2.5	33.3	1791.5	50.0	35.0	5.4	2018.5	0.0	0.0	0.0	0.0	100.0	0.0	46.7	4667.0	20.0	4.8	11.1	318.6
Crustacea, Euphausiacea, Decapoda	25.0	73.9	16.7	2263.0	75.0	0.0	62.5	4687.5	0.0	0.0	0.0	0.0	100.0	0.0	13.3	1333.0	0.0	0.0	0.0	0.0
Gastropoda	0.0	0.0	0.0	0.0	0.0	0.0	0.0	0.0	0.0	0.0	0.0	0.0	0.0	0.0	0.0	0.0	0.0	0.0	0.0	0.0

Location: *PB* Penyu Bay (Cilacap, Java), *JB* Jimbaran (Kedonganan, Bali), *F* frequency of occurrence in %, *W* weight percentage of prey in %, *N* numerical percentage of prey in %, *IRI* Index of Relative Importance. For *Mobula kuhlii*, no food items could be recorded

**Table 5:** Stomach content analyses of investigated ray species from different locations in 2009

Diet Contents	*Brevitrygon heterura *				*Brevitrygon *cf. *heterura *				*Gymnura zonura*				*Maculabatis gerrardi*				*Neotrygon caeruleopunctata*				*Rhinobatus penggali*			
	PB				PB				PB				PB				PB				PB			
	*F* (%)	*W* (%)	*N* (%)	IRI	*F* (%)	*W* (%)	*N* (%)	IRI	*F* (%)	*W* (%)	*N* (%)	IRI	*F* (%)	*W* (%)	*N* (%)	IRI	*F* (%)	*W* (%)	*N* (%)	IRI	*F* (%)	*W* (%)	*N* (%)	IRI
Teleostei	0.0	0.0	0.0	0.0	0.0	0.0	0.0	0.0	60.0	100.0	64.3	9857.1	0.0	0.0	0.0	0.0	0.0	0.0	0.0	0.0	60.0	27.4	21.4	2928.0
Cephalopoda	0.0	0.0	0.0	0.0	0.0	0.0	0.0	0.0	0.0	0.0	0.0	0.0	0.0	0.0	0.0	0.0	0.0	0.0	0.0	0.0	0.0	0.0	0.0	0.0
Crustacea, Euphausiacea, Decapoda	80.0	100.0	100.0	16,000.0	40.0	100.0	100.0	8000.0	40.0	0.0	14.3	571.4	40.0	100.0	100.0	8000.0	100.0	100.0	100.0	20,000.0	100.0	72.8	78.6	15,140.0
Gastropoda	0.0	0.0	0.0	0.0	0.0	0.0	0.0	0.0	20.0	0.0	21.4	428.6	0.0	0.0	0.0	0.0	0.0	0.0	0.0	0.0	0.0	0.0	0.0	0.0

Location: *PB* Penyu Bay (Cilacap, Java), *F* frequency of occurrence in %, *W* weight percentage of prey in %, *N* numerical percentage of prey in %, *IRI* Index of Relative Importance

**Fig. 2:**
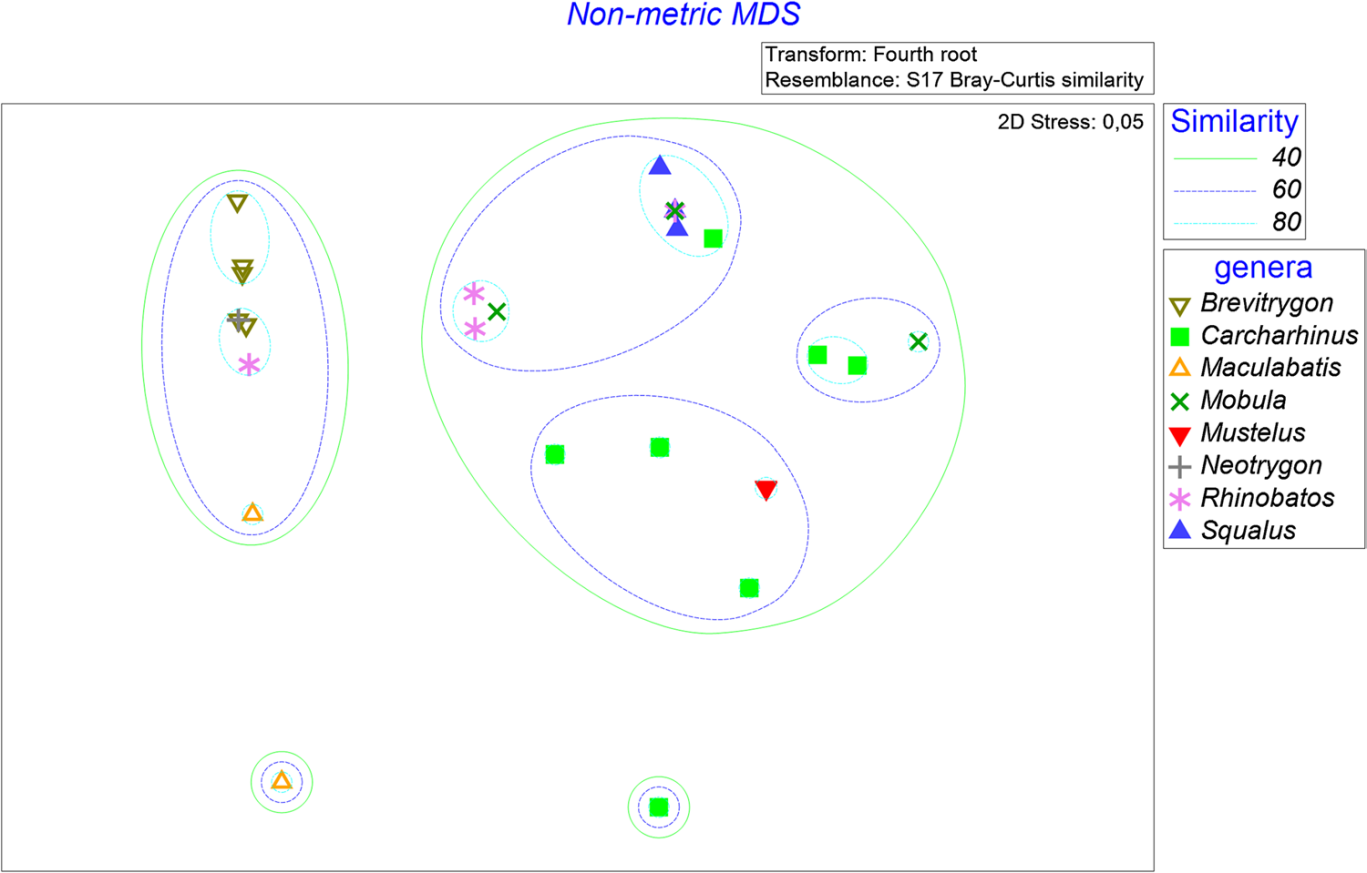
Plot of non-metric multi-dimensional scaling ordination and cluster group based on Bray–Curtis similarity of the number of individual prey found in each elasmobranch host genus

Dietary composition, in the form of prey group found in each sample, was subjected to non-metric nMDS ordination in Fig. 2. Overall, several distinctive groups were formed which indicate different compositions of their diet. The genera *Brevitrygon* and *Maculabatis* (and, in part, *Rhinobatos*) formed the most evident cluster. The *Brevitrygon* and *Maculabatis* species group was characterized by Crustacea (dominated by Euphausiacea) as their main prey item. The remaining clusters of *Carcharhinus, Mobula* and *Mustelus*, were characterized by their more variable diet across Teleostei, Crustacea and Cephalopoda. Within this group, the smaller cluster of *Carcharhinus, Rhinobatos* and *Squalus,* had cephalopods as their main prey items (Fig. 2).

## Parasite Infections

Ten different trypanorhynch species were identified from the elasmobranchs from Penyu Bay (PB), Cilacap, Java (Table 6). Most species have previously been recorded from Indonesian waters ([29]), however *Nybelinia lingualis* (Dollfus, 1929) is newly reported from this region and is described below. The single collected plerocercoid had the uncinate hooks with anterior extension of the base in the metabasal armature with the length of 13.67–14.6 μm and base length of 12.07–13.82 μm. In the basal armature, the basal hooks had the length of 8.15–10.59 μm and the base length of 8.22–10.5 μm.

**Table 6:** Isolated trypanorhynch cestodes of elasmobranch species from Penyu Bay (Cilacap, Java) in 2009

Trypanorhyncha-Species	Family	Stage	Location	Host	*n* hosts	IH	*P*%	mI	I	mA
*Dollfusiella* sp.*	Eutetrarhynchidae	A	SV	*Brevitrygon* cf. heterura**	5	1	20.0	1.0	1	0.2
Gilquinia robertsoni*	Gilquiniidae	A	SV	*Squalus megalops*	5	3	60.0	16.7	5–36	10.0
Grillotia yuniariae*	Lacistorhynchidae	PLC	SV	Squalus megalops**	5	1	20.0	2.0	2	0.4
*Mixonybelinia lepturi*	Tentaculariidae	PL	ST	*Carcharhinus* sp. I**	4	1	25.0	1.0	1	0.3
Nybelinia lingualis*	Tentaculariidae	PL	ST	Mobula kuhlii**	1	1	100.0	1.0	1	1.0
Parachristianella baverstocki*	Eutetrarhynchidae	A	SV	Maculabatis gerrardi **	5	4	80.0	9.0	2–27	7.2
	Eutetrarhynchidae	A	SV	Brevitrygon heterura**	5	1	20.0	2.0	2	0.4
	Eutetrarhynchidae	A	SV	Neotrygon caeruleopunctata **	2	1	50.0	3.0	3	1.5
Parachristianella indonesiensis*	Eutetrarhynchidae	A	SV	Maculabatis gerrardi**	5	1	20.0	4.0	4	0.8
Parachristianella monomegacantha*	Eutetrarhynchidae	A	SV	Maculabatis gerrardi**	5	4	80.0	9.0	2–27	7.2
	Eutetrarhynchidae	A	SV	Rhinobatos penggali**	5	5	100.0	29.2	17–39	29.2
	Eutetrarhynchidae	A	SV	Brevitrygon heterura**	5	1	20.0	18.0	18	3.6
	Eutetrarhynchidae	PLC	M	Rhinobatos penggali**	5	2	40.0	15.5	1–30	6.2
*Parachristianella* spp.*	Eutetrarhynchidae	A	SV	Maculabatis gerrardi**	5	4	80.0	10.0	2–27	8.0

*A* adult, *IH* infected hosts, *L* larva, *PC* plerocercoid, *PL* postlarva (immature specimens), *PLC* plerocercus, *ST* stomach, *SV* spiral valve, *I* Intensity, *mA* mean Abundance, *mI*: mean Intensity, *P*% prevalence in percent

*new locality record for this region

**new host record

Other characters were a tentacle width (TW) basal = 50 µm, TW metabasal = 43 µm and basal swelling absent. Tentacle sheath straight, Tentacle sheath width = 18. Half spiral row (hsr) = 7 basal, hsr = 7 metabasal. This character combination indicated conspecificity to this most commonly reported trypanorhynch of rays, especially from the Atlantic Ocean.

Two species, *Dollfusiella* sp. (Campbell & Beveridge, 1994) from *Brevitrygon* cf. *heterura* of Penyu Bay, Cilacap, Java and *Prochristianella* sp. (Dollfus, 1946) from *M. gerrardi* from Jimbaran, Kedonganan, Bali, were not identified to species level. Both had an unrecognized character combination, especially of the tentacular armature and might represent new species.

Within the families Gilquiniidae (Dollfus, 1935), Lacistorhynchidae (Guiart, 1937) and Pterobothriidae (Pintner, 1931) only one species was found respectively, while two species of Tentaculariidae (Poche, 1926) and four species of the family Eutetrarhynchidae (Guiart, 1927) were identified from Java. Rays and sharks from the Balinese sampling included four different parasite species (Table 7); three species belonged to the Eutetrarhynchidae and one species to the Tentaculariidae*.*

**Table 7:** Isolated trypanorynch cestodes of investigated elasmobranch species from Jimbaran (Kedonganan, Bali) in 2009

Trypanorhyncha-Species	Family	Stage	Location	Host	*n* hosts	IH	*P*%	mI	*I*	mA
*Dollfusiella* sp.*	Eutetrarhynchidae	A	SV	Maculabatis gerrardi**	1	1	100	2	2	2
Kotorella pronosoma*	Tentaculariidae	A	ST	*Maculabatis gerrardi*	1	1	100	2	2	2
Parachristianella baverstocki*	Eutetrarhynchidae	A	SV	Maculabatis gerrardi**	1	1	100	12	12	12
*Prochristianella* sp.*	Eutetrarhynchidae	A	SV	Maculabatis gerrardi**	1	1	100	3	3	3

*A* adult, *IH* infected hosts, *ST* stomach, *SV* spiral valve

*new locality record

**new host record

**Fig. 3:**
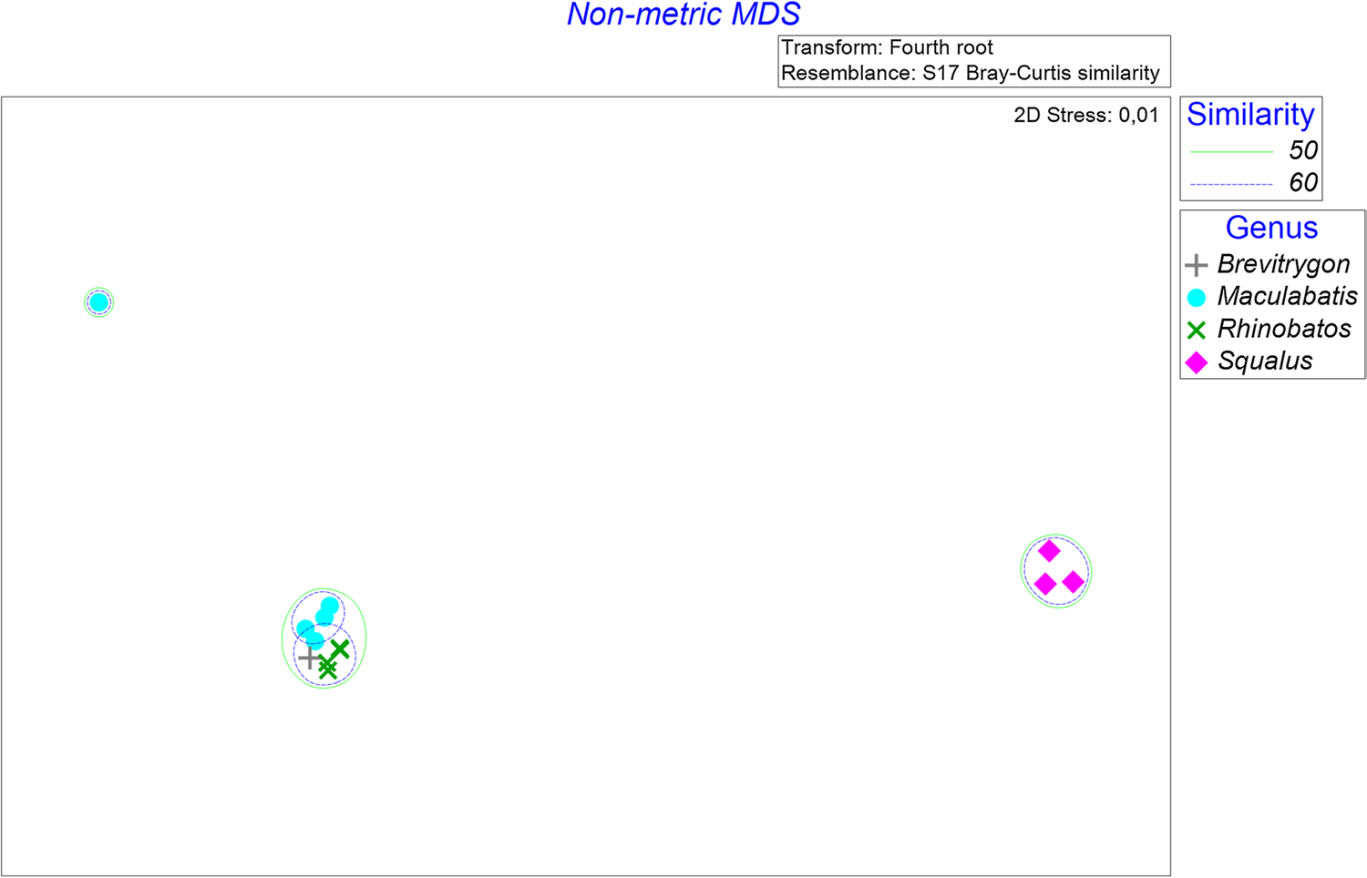
Plot of non-metric multi-dimensional scaling ordination and cluster group based on the Bray–Curtis similarity of parasite species abundance in each elasmobranch host genera

*Parachristianella monomegacantha* (Kruse, 1959) (from *R. penggali*) and *P. baverstocki* (Beveridge, 1990) (from *M. gerrardi*) showed the highest prevalence (100% each) and mean intensities (29.2 and 12.0), respectively, in rays from each collection location (Table 6, Table 7). *Gilquinia robertsoni* (Beveridge, 1990) (from *S. megalops*) showed the highest prevalence (60%) and mean intensity (16.7) in sharks collected from Java (Table 6). None of the sharks collected from Bali were infected with cestodes. For *C. sorrah, Gy. zonura* and *Mustelus* cf. *manazo,* no parasites were recorded. Analysis of multi-dimensional scaling provided information on similarities of parasites of host samples collected from Penyu Bay (Central Java) and Jimbaran (Bali). The analysis resulted in two major groups, which is likely to be formed based on host genus level. One group is formed by the host genera *Brevitrygon, Maculabatis,* and *Rhinobatus*, implying a closer relationship of the parasitological communities of these three genera than those of *Squalus*, which formed the other cluster. However, one group of the *Maculabatis* samples taken from Jimbaran formed a different cluster, implying dissimilarities for *Maculabatis* from Penyu Bay, due to the presence of the parasite species *Kotorella pronosoma*, (Euzet & Radujkovic, 1989) and genera *Dollfusiella* sp. and *Prochristianella* sp. The genera *Brevitrygon*, *Maculabatis* and *Rhinobatus* from Penyu Bay shared their similarity, especially in terms of *Parachristianella baverstocki* and *P. monomegacantha* and *Parachristianella* spp. The cluster of the genus *Squalus* is likely to be formed due to the presence of the cestode *Gilquinia robertsoni* and/or the absence of the genera *Parachristinella* (Fig. 3).

## Interactions between Host Diet and Parasite Infections

The teleost dominated diet of the sharks *Carcharhinus* sp. 1 (100%) and *Squalus megalops* (95.2% Teleostei in stomach content in weight percent) is reflected in the parasite infection. *Gilquinia robertsoni* and *Grillotia yuniariae* (Palm, 2004) were detected in *S*. *megalops* and *Mixonybelinia lepturi* (Palm, 2004) in *Carcharhinus* sp. 1. The larval stages of *Gr*. *yuniariae* and *Mi*. *lepturi* are known from many teleost fishes [29].

*Parachristianella monomegacantha*, is amongst the most abundant and frequent parasite species within this study. Its transmission pathway and life cycle include Crustacea (Copepoda) as 1st intermediate hosts and Crustacea (shrimps, possibly euphausiids and crabs) as 2nd intermediate hosts before finally infecting rays (life cycle described in Mudry and Dailey [48]). It is also known that the life cycle for species of *Dollfusiella*, *Parachristianella* and *Prochristianella* include Crustacea as intermediate hosts [49–51]. Owing to the high infection rates observed, Crustacea must play an important role within the feeding ecology of the investigated hosts (*Brevitrygon heterura* (100%), *Maculabatis gerrardi* (100%), *Neotrygon caeruleopunctata* (100%), and *Rhinobatos penggali* (72.8% Crustacea in stomach content in weight percent)) of these trypanorhynch cestodes.

## Discussion

The results of the present study help to fill gaps in general ecological knowledge, like feeding preferences, for several elasmobranch species from Indonesian waters. The results also combine the trypanorhynch fauna with reported dietary items to help determine the routes of transfer of trypanorhynch cestodes to these elasmobranchs. We are aware that this study, similar to Palm *et al*. [26], relied on opportunistic sampling of a number of elasmobranch species, with limited possibilities for data extrapolation. However, as any biological and ecological data about free-living sharks and rays is scarce, all available resources concerning feeding ecology and ecological preferences, health status and parasitism, must be considered.

The shark and ray species examined in the present study were mainly benthic species based on their stomach contents. In general, teleost fishes and cephalopods, including squid and octopus, were the major diet of the sharks, while the rays had diets dominated by crustaceans. The examined sharks and rays mostly had similar ranges of total lengths (less than 100 cm) and preyed upon the same benthic organisms in the same regions [52, 53]. Many of the elasmobranch species studied have limited zoogeographical distribution ranges, being endemic in Indonesia waters or specifically endemic to the southern waters of Java and Bali. On the other hand, *Mobula kuhlii* and *Squalus megalops* are usually rarely caught and landed in coastal waters. *Squalus megalops* is a deep-sea shark from the Central Indo-Pacific, while *M. kuhlii* is a pelagic ray species; both of these species were able to be included in this study due to the landing site, which was located on the edge of the Indian Ocean.

The studied parasite community and feeding ecology revealed certain patterns within the examined group of sharks and rays. The analysis of the feeding ecology showed several clusters with partially overlapping host groups. In particular, the trypanorhynch parasite community of *S. megalops* showed a distinct separation as compared to those of the other elasmobranchs, which clustered together. This clear separation can be explained by the ecology (see above), as well as other factors which may drive the differences. *Squalus megalops* occurs in depths up to 732 m [54], and it has a different range compared with the other sharks which are commonly known as coastal species. According to Palm *et al*. [26], influencing factors in addition to the diet may be depth orientation of the fishes, their habitat or their phylogeny/taxonomic classification. Palm *et al*. [26] stated in their study that the most remarkable relationship exists between the habitat type and the trypanorhynch assemblage. As Palm *et al*. [26] stated, transmission through the marine food web (via predation) and an unambiguous identification in the final (sharks and rays) and intermediate hosts (teleosts, other marine invertebrates) allow conclusions to be made on the feeding biology of the host. Their study demonstrated that nMDS of the elasmobranch parasitic Trypanorhyncha was a useful tool for investigating parasites as indicators of the host biology in marine ecosystems, especially depth distribution, diet, and habitat type were the major influencing factors. This seems to be the case also in the present study, where *S. megalops* clustered separately to the other sampled elasmobranch species.

Information on parasitic diseases, or other infections, and epidemiology and infection parameters are scarce on free-living elasmobranchs. Cestodes are transferred according to the host’s feeding ecology throughout all trophic levels. Analyses of stomach contents and parasites in combination can be of further assistance to understand marine food webs and diet availabilities in different habitats, for a variety of host species and their ecology.

The shown dissimilarities in the present study might be either referred to a different degree of pollution or variances in environmental conditions in general. In Penyu Bay these differences might be caused by the nearby city Cilacap and its industries or are resulting from a different feeding ecology, e.g., differences of the examined cartilaginous elasmobranchs, reflecting the different roles of intermediate host for the occurrence of the trypanorhynch cestodes at the sampling sites. Our results demonstrate that the trypanorhynch fauna varies within the studied vulnerable and endangered elasmobranchs, while the feeding ecology is an influencing factor for their composition. Such studies will shed more light onto the scarce knowledge on the elasmobranchs ecology and can be seen as a future baseline for the health status and conservation studies of Indonesian sharks and rays.

## Data Availability

Data would be made available on reasonable request.
